# The cardiac niche role in cardiomyocyte differentiation of rat bone marrow-derived stromal cells: comparison between static and microfluidic cell culture methods

**DOI:** 10.17179/excli2018-1539

**Published:** 2018-08-01

**Authors:** Seyed Ahmad Vaez, Somayeh Ebrahimi-Barough, Masoud Soleimani, Sedighe Kolivand, Saeed Farzamfar, Seyed Hossein Ahmadi Tafti, Mahmoud Azami, Farshid Noorbakhsh, Jafar Ai

**Affiliations:** 1 Department of Tissue Engineering and Applied Cell Sciences, School of Advanced Technologies in Medicine, Tehran University of Medical Sciences, Tehran, Iran; 2 Department of Hematology, Faculty of Medical Sciences, Tarbiat Modares University, Tehran, Iran; 3 Department of Medical Biotechnology, School of Advanced Technologies in Medicine, Tehran University of Medical Sciences, Tehran, Iran; 4 Research Center for Advanced Technologies in Cardiovascular Medicine, Tehran Heart Center, Tehran University of Medical Sciences; 5 Department of Immunology, Faculty of Medicine, Tehran University of Medical, Sciences, Tehran, Iran

**Keywords:** cardiomyocyte isolation, microfluidic system, bone marrow-derived mesenchymal stromal cells (BMSCs), differentiation, niche, co-culture

## Abstract

Due to the restricted potential of the heart to regenerate its damaged region, stem cell therapy is a promising treatment modality for myocardial infarction. It has been shown that incubation of bone marrow-derived stromal cells (BMSCs) with initial steps of cardiac differentiation *in vitro*, can have a significant effect on their therapeutic potential to treat myocardial infarction. Based on these well-established principals we were encouraged to study the direct co-culture of rat BMSCs with neonatal mouse almost pure cardiomyocytes (APCs) and cardiac niche cells (CNCs) in static 2D and microfluidic cell culture systems. Our results showed that the difference regarding the beating rate in isolated APCs and CNCs in both 2D and the microfluidic system was not statistically significant for 30 days. No beat rate could be observed in induced BMSCs in all groups during experiment time. Except for BMSCs cultured alone in both experimental culture conditions, data obtained from Real-time PCR analysis showed that differentiated BMSCs in all co-cultured groups expressed GATA4, Nkx2.5, CX43, cTnI, cTnT, and β-MHC during 4 weeks. BMSCs demonstrated a higher expression of these cardiac factors in microfluidic chips than those co-cultured in 24 well plates. Moreover, immunocytochemistry (ICC), also revealed the GATA4 expression in differentiated BMSCs in all co-cultured groups. It was found that, when combined with shear stress, co-culture with cardiomyocyte can differentiate BMSCs significantly toward cardiomyocyte rather than co-culture alone.

## Introduction

Myocardial infarction (MI) afflicts millions of patients annually and this disease accounts for a high mortality rate worldwide (Xu and Guan, 2016[31]). MI is accompanied by inflammation, loss of cardiomyocyte, fibrous scar formation, and a gradual weakening of the myocardium functions (Xu and Guan, 2016[31]). Although medical approaches are accessible for treating MI, these treatments are not satisfactory (Furtado et al., 2016[8]).

Due to the restricted capacity of the heart to regenerate its injured tissue after MI, stem cell therapy has been considered a potential treatment modality (Antonitsis et al., 2007[1]). Despite the fact that some kinds of stem cells may be suitable for cell-based therapy in heart failure, bone marrow-derived stromal cells (BMSCs) are appealing since they can be simply extracted and expanded in culture (Antonitsis et al., 2007[1]). It has been shown that BMSC-based therapy and tissue engineering for heart failure is feasible and safe (Chen et al., 2017[5]). In addition, BMSCs were shown to have some other vital biological advantages including angiogenesis, immune modulation, and anti-apoptotic activities (Chen et al., 2017[5]). Furthermore, the multipotent differentiation of BMSCs endows them with the capability to differentiate into different cell lineages such as chondrocyte, osteocyte and adipocyte-like cells (Chen et al., 2017[5]). 

Differentiation potential of BMSCs into cardiomyocyte phenotype have been proved both *in vitro* and *in vivo* (Madigan and Atoui, 2018[17]; Wollert et al., 2004[29]). These studies emphasized that BMSCs can express cardiac markers, present sarcomeric structures, and produce electro-mechanical activities. Moreover, there are clinical trials showed that BMSCs can reduce infarction sites, ameliorate left ventricular ejection fraction, and improve angiogenesis (Madigan and Atoui, 2018[17]; Wollert et al., 2004[29]). On the other hand, some authors believe that only a minor portion of engrafted cells would differentiate into cardiomyocytes (Antonitsis et al., 2007[1]), however, several reports mentioned that BMSCs do not transdifferentiate into functional cardiomyocytes (Rose et al., 2008[21]; Siegel et al., 2012[24]). The researchers believe that the mechanism by which transplantation of BMSCs exert their ameliorative effects on heart function after MI is due to secretion of immunomodulatory and angiogenic factors, the initiation of paracrine signaling cascades, and activation of endogenous cardiac stem cells (Ding et al., 2015[6]). Based on the above-mentioned reasons, there is a possibility that if BMSCs are subject to initial steps of cardiac differentiation *in vitro* prior to transplantation, the final engraftment and clinical results might be improved (Antonitsis et al., 2007[1]). 

Previous studies showed that chemical agents like 5-azacytidine can induce BMSCs to differentiate into cardiomyocytes (Behfar et al., 2010[3]; Makino et al., 1999[18]). Co-culture is another way to differentiate stem cells into cardiomyocytes. It seems that co-culture of BMSCs with cardiomyocytes is much closer to the natural condition of the body than other approaches (He et al., 2010[11]). Co-culture is also suitable for the assessment of physical contact and soluble factors effects on differentiation yield (Bogdanowicz and Lu, 2013[4]). The *in vitro* co-culture of BMSCs with cardiomyocytes and other cells located in the heart native niche can partially simulate transplantation of BMSCs into the heart (He et al., 2010[11]). The heart niche consists of several cells; only about 20-40 % of the cells in the heart are cardiomyocytes and myocardium is mainly composed of cardiac fibroblast (Souders et al., 2009[26]). The most important roles of fibroblasts are to remodel extracellular matrix (ECM) and transmit mechanical forces produced by cardiomyocyte to the ECM and other cells (Murthy et al., 2006[19]). In this regard, emerging factual information shows an integral role of fibroblasts as a crucial participant in reaction to injury and also as a key player in normal cardiac function (Kakkar and Lee, 2010[15]). 

On the other hand, advanced organs-on-a-chip technology, in recent times, has simulated tissue models inside a microfluidic system mimicking the cardiovascular system (Zhang et al., 2015[33]). The most important challenge for researchers in tissue engineering is to reestablish a microenvironment in order to induce cells differentiation and organize them in a well-arranged functional tissue (Verhulsel et al., 2014[28]). Cells receive several spatiotemporal signals from surrounding niche, which may impact their activities (Bogdanowicz and Lu, 2013[4]). As an alternative, the microfluidic platform may improve the investigation of cellular behavior *in vitro* since it supplies tools for mimicking *in vivo*-like microenvironments (Huang and Lee, 2007[12]). It seems that microfluidic cues together with cardiac niche cells may have beneficial effects on BMSCs differentiation towards cardiomyogenic lineage.

This study was aimed to assess the *in vitro* ability of rat BMSCs to myogenic conversion in co-culture with mouse isolated almost pure cardiomyocytes (APCs) and cardiac niche cells (CNCs) in static 2D and microfluidic cell culture systems. Consequently, the goal of this study was to evaluate the potential roles of the cardiac niche cells as well as shear stress in the cardiac regeneration by contributing to the differentiation of BMSCs into cardiomyocytes.

## Materials and Methods

### Chemicals

All tissue culture media and supplements were purchased as follows: penicillin-streptomycin, trypsin-EDTA, Dulbecco's Modified Eagle Medium (DMEM), fetal bovine serum (FBS), and collagenase type II (Gibco); bovine serum albumin (BSA), Bromodeoxyuridine (BrdU), insulin, 3-isobutylmethylxanthine (IBMX), β- glycerophosphate, ascorbic acid, dexamethasone, and indomethacin (Sigma); monoclonal antibodies against CD34 (Abcam), CD44, CD90 (Biolegend), and CD45 (Thermofisher); rabbit anti-rat GATA4 primary antibody (ab84593), Donkey F(ab')2 Anti-Rabbit IgG H&L (PE) preadsorbed (ab7007); RNX- Plus (SinaClon, Iran), cDNA synthesis kit (TaKaRa, Japan), RealQ Plus 2x Master Mix Green (Amplicon, Denmark); SU-8 2050 (MicroChem, Newton MA, USA), PolyDimethylSiloxane (PDMS), and curing agent (Dow Corning, USA). 

### Microfluidic device fabrication

**Figure 1:**
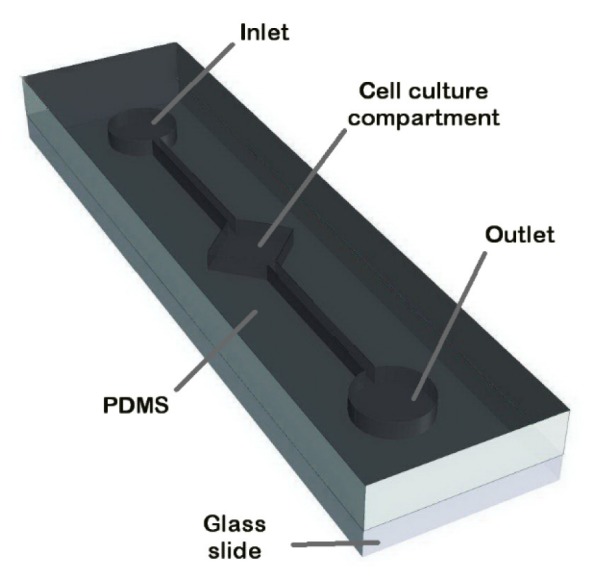
Schematic illustration of the fabricated microfluidic device utilized in this study

The microfluidic pattern was designed by AutoCAD software and was printed on a transparency film as a photomask. A silicon wafer was spin coated with 25 µm thick SU-8 2050 and was exposed to UV light to produce master mold via standard microfabrication (soft lithography) process (Siltanen et al., 2016[25]). Briefly, PDMS was mixed with crosslinker resin (10:1 (w/w) ratio) on the wafer template and baked at 85 °C for 2 h. The formed PDMS structures with a thickness of 2 mm were plasma-oxidized using corona surface treater (Electro-Technic Products, INC) for 10 min for permanent bonding to glass coverslips. Afterward, the microfluidic platform was sterilized using UV exposure for 20 min before connecting to the syringe pump (Figure 1(Fig. 1)).

### BMSCs isolation, characterization, and GFP transduction

Isolation and primary culture of BMSCs from 6-8 weeks Wistar rats were performed according to a previously described protocols with minor modifications (Ding et al., 2015[6]). Briefly, rats were sacrificed by intraperitoneal injection of 75 mg/kg ketamine followed by cervical dislocation, femurs and tibias were aseptically removed and soaked in 70 % ethanol for 10 minutes on ice. Under the hood, both ends of each bone were cut and bone marrow was then flushed out using basal medium including DMEM supplemented with 10 % FBS, 100 µg ml^-1^ penicillin and 100 µg ml^-1^ streptomycin. The harvested cells were plated and cultured in the basal medium at 37 °C in a humidified atmosphere of 5 % CO_2_. After 24 hr, the medium was slowly changed, and suspended cells were discarded. The medium was replenished every 3 days. After 2 weeks of initiating culture, upon reaching 80 %-90 % confluency, the cells were subcultured in a 1:2 ratio with 0.25 % trypsin containing 0.02 % ethylenediaminetetraacetic acid (EDTA) for 2 min at room temperature and all lifted cells were cultured in a 25-cm^2^ flask. For all the subsequent experiments, cells from the 3-4 passage were used. 

For cell phenotypic characterization, the flow cytometry analysis was carried out by staining the cells with the monoclonal antibodies against CD34, CD44, CD45, and CD90, according to the provided protocol (He et al., 2010[11]). To investigate the multilineage differentiation potential of BMSCs, standard osteo- and adipocyte differentiation procedures were performed (Granéli et al., 2014[9]). For osteogenesis, cells were treated with 10 mM β-glycerophosphate, 200 μM/l ascorbic acid, and 0.1 μM dexamethasone for 3 weeks. Afterward, cells were fixed with 4 % paraformaldehyde for 30 minutes and stained with Alizarin Red. For adipogenesis, cells were maintained in the medium containing 10 μM insulin, 0.5 mM Isobutylmethylxanthine (IBMX), 1 μM/l dexamethasone, and 50 μM/l indomethacin for 3 weeks and then stained with Oil Red. 

Passage 3-4 BMSCs with approximately 50 % confluency in a T25 cm^2^ flask was used for transduction with lentiviral green fluorescence protein (GFP) according to a previously described protocol (Hatzistergos et al., 2010[10]). Briefly, the media was exchanged with the determined volume of transduction media (DMEM with 10 % FBS plus 4 μg/mL polybrene and 10 μg/L of unconcentrated viruses) at 37 °C. The media were changed after 12 h. Uninfected BMSCs were eliminated with 2 μg/ml puromycin starting from 48 h after transduction. Media containing Puromycin was changed every day for 4 days.

### Cardiac cells isolation 

APCs and CNCs isolation were carried out according to previously described methods with minor modifications (Vandergriff et al., 2015[27]). In short, newborn mouse pups (1-3 days old) were euthanized humanely, the chest was opened along the sternum, the heart was excised, the atrium was removed, and ventricle was minced with a curved scissor on ice and digested by 4 % collagenase type II in Leibovitz's L-15 Medium for 20 min with gentle shaking. To remove noncardiomyocyte cells, centrifugation was performed at 1000 rpm for 5 min. For further purification of APCS, 90 min pre-plating was performed at 100,000 cell/cm^2^. Non-adherent cells were collected and seeded in gelatin-coated plates. CNCs was used as whole cardiac isolated cells without any non-myocyte cell removal. BrdU (1 μg/mL) was added to all the samples after 12 hrs and incubated for 24 hrs to inhibit fibroblasts and other nonmyocytes proliferation. This method permitted us to obtain attached cardiac myocytes representing spontaneous contractility throughout the entire experiment time.

### Beat rates counting of APCs and CNCs

After cell isolation, 1×10^5 ^APCs and CNCs were seeded in 24 well plates separately (each sample was triplicated). All groups were maintained at 37 °C in a humidified air containing 5 % CO_2_ in the basal medium which was changed every 3 days. To study the potential effects of fibroblasts and other cardiac niche cells on cardiomyocyte beats, each well was observed under Labomed tcm400 inverted microscope on days 1, 3, 5, 10, 15, 20, 25, and 30 and cardiomyocyte beat rates were counted. 

### Differentiation protocols 

To investigate the capability of cardiomyocytes and other cardiac niche cells to differentiate BMSCs into cardiomyocytes, this study was conducted based on 1:1 co-culture of BMSCs with: (1) APCs in cell culture plate, (2) APCs in microfluidic system, (3) CNCs in cell culture plate, (4) CNCs in microfluidic system. The cells were seeded at a density of 2×10^5^/cm^2^. After 12 h, cells were treated with 1 µg/ml BrdU for 24 h to inhibit cell proliferation in both 2D and microfluidic cell culture systems. Cells were then cultured in basal medium at 37 °C in a humidified incubator containing 5 % CO_2_. The half medium was replaced every 3 days with the fresh basal medium in 2D culture. Under the following equation, the shear stress (τ) of 10 dyn/cm^2^ with the basal medium was applied to the microfluidic cell culture device as described previously (Huang et al., 2010[13]).


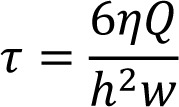


where τ indicates the applied shear stress (dyn/cm^2^), *η* stands for the dynamical viscosity of DMEM at 37 °C (0.84 Pa-s), Q is the volumetric flow rate (cm^3^/s), h and w are the height and width of the channels (cm), respectively. Also, to examine the potential of each cell culture condition to differentiate BMSCs into cardiomyocyte without any other differentiating factor, BMSCs was cultured alone in both cell culture conditions. Each 2D and microfluidic culture experiments was performed three times to validate the results. During differentiation, cardiac gene expression was evaluated on weeks 2 and 4.

### Quantitative Real-Time Reverse Transcription Polymerase Chain Reaction (Real-Time PCR) 

The expression of cardiac-specific genes including NK2 transcription factor related locus 5 (Nkx2.5), GATA binding protein 4 (GATA4), beta-myosin heavy chain (β-MHC), cardiac troponin I3 (TNNI3 or cTnI), cardiac troponin T2 (CTNT2 or cTnT), and gap junction protein alpha 1 or connexin 43 (GJA1 or CX43) were examined in different groups using specific rat real-time PCR primers (Table 1(Tab. 1)) as described previously (Rose et al., 2008[21]). Briefly, total RNA from APCs, APCs-BMSCs, CNCs, and CNCs-BMSCs in both cell culture conditions were extracted during differentiation on weeks 2 and 4 using the RNX-Plus solution. RNA quantity and purity was assessed by NanoDrop ONE (Thermoscientific) and the integrity was confirmed by gel electrophoresis. The absence of contaminating genomic DNA was confirmed by the no reverse transcriptase control (-RT) for each sample. Furthermore, cDNA synthesis kit was used to prepare cDNA. Real-Time PCR was performed with the rotor gene Q series (Qiagen, USA) in a reaction volume of 10 μl. Briefly, 5 μl of master mix, 1 μl of primer sequences, 3 μl PCR-grade H_2_O and 1 μl of template cDNA were added to each sample. The reaction was carried out as follows: enzyme activation at 95 °C for 15 min, initial denaturation at 95 °C for 15 s, annealing temperature at 60 °C for 30 s, and extension at 72 °C for 30 s, followed by 40 cycles. The relative expression ratio of mRNA was calculated by the 2^−ΔΔCT^ method. Data are presented as fold changes of these genes relative to the expression of glyceraldehyde-3-phosphate dehydrogenase (GAPDH) as a housekeeping gene.

### Immunocytochemistry (ICC) analysis

To evaluate the differentiation potential of BMSCs to myogenic lineage by different co-culture methods, we performed ICC analysis against specific cardiomyocyte proteins according to a previously described protocol (Shi et al., 2016[23]). Briefly, after 14 days of co-culture, cells were washed 3 times with PBS and fixed for 10 min with 4 % paraformaldehyde. Cells were incubated afterward for 30 min with 0.2 % Triton X-100 and then blocked with 10 % BSA. Cells were incubated with primary antibody overnight at 4 °C. Primary antibody used for cardiomyocyte were mAb's for GATA4 at a dilution of 1:50. After three washes with PBS, cells were incubated with secondary goat anti-mouse, FITC-conjugated IgG1 antibody (1:100) for 1 hour at room temperature. Nuclei were stained with 4',6-diamidino-2-phenylindole (DAPI). The images were obtained using a Nikon Eclipse 600 microscope using the NIS-Elements AR analysis software (Nikon Instruments Inc., Tokyo, Japan). 

### Statistical analysis

Data were analyzed using one-way analysis of variance (ANOVA) followed by Tukey's post hoc test to determine any difference between groups. The SPSS software version 20.0 (SPSS, Inc, Chicago, IL, USA) was used for data analysis. All data are expressed as a mean ± standard deviation of the mean (mean ± SD); values of p < 0.05 were considered to be statistically significant. 

## Results

### BMSCs isolation and characterization

**Figure 2:**
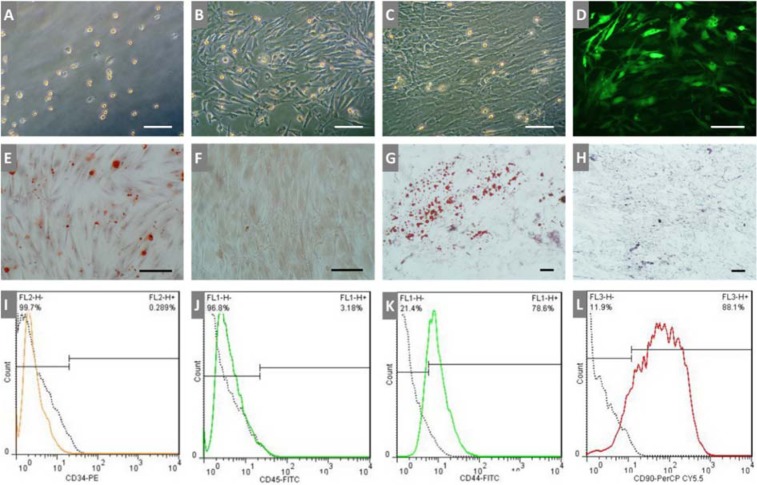
BMSCs isolation and characterization. (A-C): morphological features of rat BMSCs; (A): 2^nd ^day of isolation, bright, round, and small cells adhered to plate floor; (B): 14^th^ day of isolation, spindle-shaped morphology; (C): 21^st^ day of isolation, cells became more confluent; (D): GFP labeled BMSCs. (E-H): differentiation of BMSCs toward osteo- and adipocytes. (E): mineralizing cells stained with alizarin red; (F): negative control for alizarin red staining; (G): oil red staining represents oil accumulation in the cells; (H): negative control for oil red staining. (I-L): flow cytometry analysis of BMSCs surface markers. Scale bar = 100 μm

BMSCs from 6-8 weeks old male rats were successfully cultured, expanded and used for subsequent tests. After washing with PBS at day 2-3 to remove nonadherent cells, the remaining round and small adherent cells formed colonies and then changed to polyhedral and longer morphology gradually in the next days (Figure 2 A-D(Fig. 2)). Only BMSCs from the 3-4 passage were used for all the subsequent experiments. It has been shown that under proper conditions, BMSCs can differentiate into several lineages such as adipocyte, chondrocyte, and osteocyte. Induction of cell differentiation was performed with β-glycerophosphate, ascorbic acid, and dexamethasone, or with insulin, IBMX, dexamethasone, and indomethacin into an osteogenic (Figure 2 E, F(Fig. 2)) or adipogenic (Figure 2 G, H(Fig. 2)) lineage, respectively. The flow cytometry (BD FACSCalibur™) report showed that the isolated BMSCs were negative for CD34 and CD45, and positive for CD44 and CD90 (Figure 2 I-L(Fig. 2)).

### APCs and CNCs isolation

**Figure 3:**
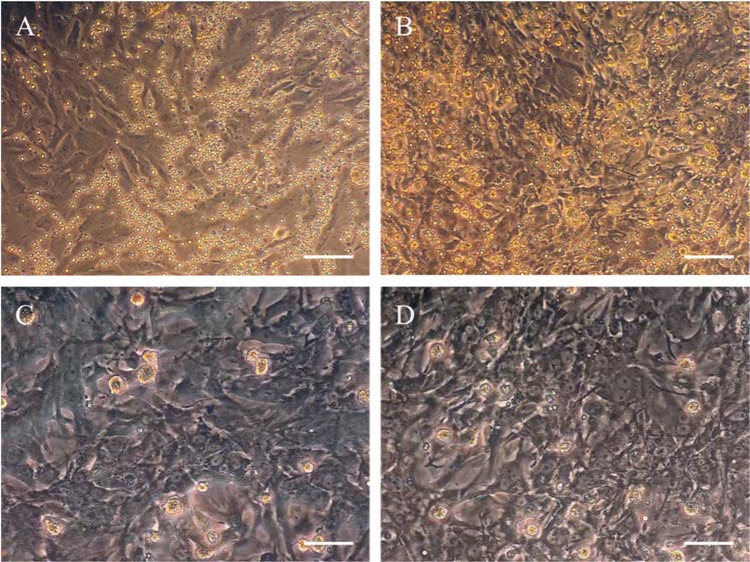
APCs and CNCs isolation. (A, B): the second day of isolation, cardiomyocytes and other adherent cells adhered to the bottom of the plates while red blood cells (RBCs) and dead cells floated; (C, D): after 2 weeks of isolation, cardiomyocytes and other cells expanded in the culture. (A, C): APCs, (B, D): CNCs. Scale bar = 100 μm

To isolate APCs from non-myocyte cells, centrifugation and preplating were performed. CNCs were used as whole cardiac isolated cells without any nonmyocyte cell removal. The method resulted in cardiomyocytes which could spontaneously beat throughout the entire cultivation period (Figure 3(Fig. 3)). 

### APCs and CNCs beating rates assessment

**Figure 4:**
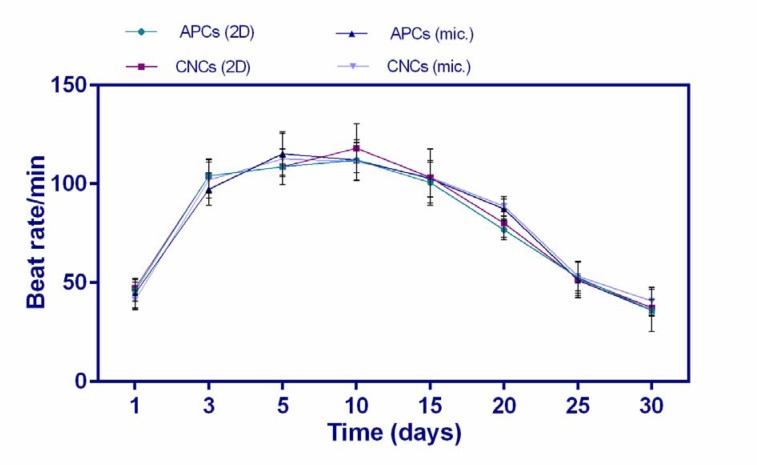
Beating rates comparison among groups on defined days. 2D: static 2D culture; mic.: microfluidic cell culture

Obtained data revealed that beat rates diminished from the 10^th^ day of the study to end of the experiment in all groups. Statistically, no significant difference was observed in beating rates among groups (Figure 4(Fig. 4)). 

### Real-Time PCR results of BMSCs cardiac-specific gene expression 

Cardiac gene expression level of rat BMSCs co-cultured with mouse APCs and CNCs was analyzed during differentiation on weeks 2 and 4. To avoid detection from mouse cardiomyocytes, rat-specific real-time PCR primers were used (Table 1(Tab. 1)) to detect the expression of Nkx2.5, GATA4, cTnI, cTnT, CX43, and β-MHC which were normalized to GAPDH expression. 

**Figure 5:**
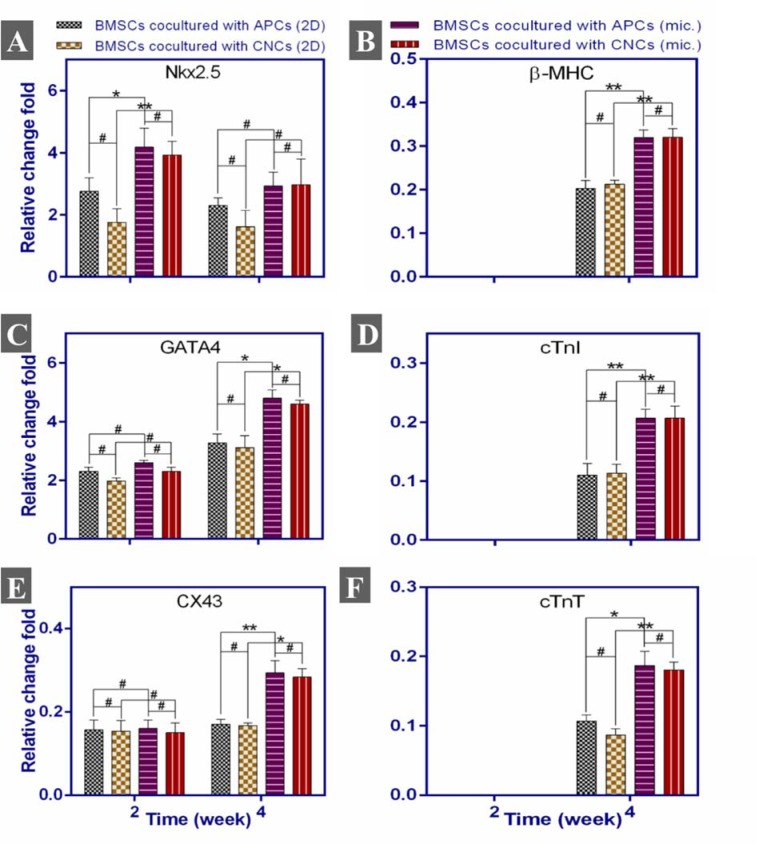
The Nkx2.5 (A), β-MHC (B), GATA4 (C), cTnI (D), CX43 (E), cTnT (F) gene expression in BMSCs co-cultured with APCs and CNCs for 2 and 4 weeks. The expression of cardiac-specific genes was seen only on week 4. #: no differences between groups, *:p<0.05, **:p<0.01, 2D: 2D cell culture system, and mic.: microfluidic cell culture device

Attained data from real-time PCR analysis in static 2D and microfluidic cell culture systems showed that GATA4 and CX43 had an increasing tendency in gene expression until week 4; however Nkx2.5 expression, was greatest at week 2 (Figure 5(Fig. 5)). The specific cardiac gene expression of cTnI, cTnT, and β-MHC also was detectable only in week 4. The cardiac gene expression, however, was not observed in BMSCs cultured alone in both static 2D and microfluidic chip during experiment time.

### ICC outcomes

**Figure 6:**
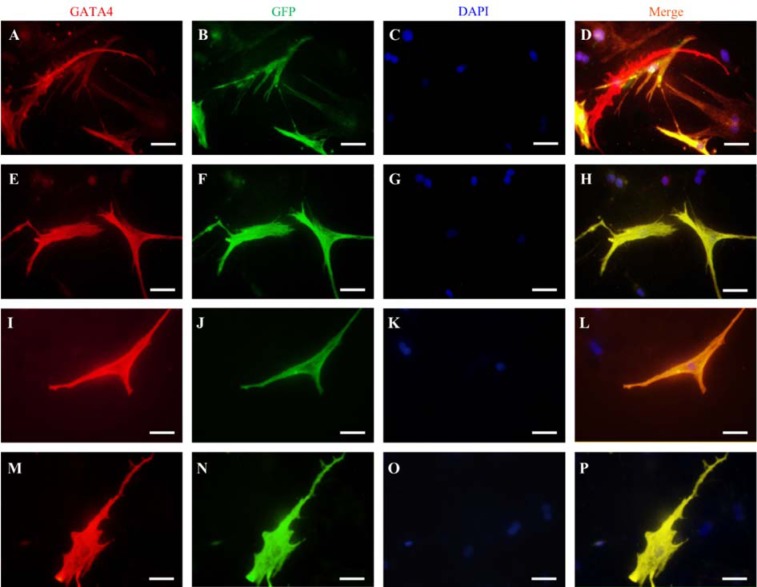
Immunofluorescence staining of GATA4 (B and E) was accomplished to exhibit cardiomyocyte gene expression in GFP-labeled BMSCs (B, F, J, and N). Nuclei were stained with DAPI (C, G, K, and O). Merged images (Merge) are shown in (D, H, L, and P). This figure shows BMSCs in co-culture with APCs in 2D (A-D), CNCs in 2D (E-H), APCs in the microfluidic system (I-L), and CNCs in the microfluidic system (M-P). This figure illustrates that APCs and CNCs could induce BMSCs to express GATA4 in both static 2D and microfluidic system. Scale bar: 20 μm

All ICC stainings were performed on day 14. Co-culture with APCs and CNCs upregulated cardiac marker GATA4 in GFP+ BMSCs in all groups (Figure 6(Fig. 6)). There was no GATA4 expression in BMSCs cultured alone without any differentiating factor in both culture conditions.

## Discussion

Stem cell therapy is an emerging promising treatment approach for cardiovascular diseases. Recent studies are attempting to discover stem cells with higher potential for the treatment of MI (Ding et al., 2015[6]). The safety and efficiency of BMSCs to treat MI have been shown in animal models (Chen et al., 2017[5]). BMSCs transplantation has demonstrated advantageous results regardless of their possible therapeutic effects; either by differentiation into cardiomyocyte-like cells or acting via paracrine signaling (e.g., by increasing angiogenesis) (He et al., 2010[11]).

Previous studies have failed to show the considerable conversion of BMSCs to myogenic lineage *in vivo *(Schäfer and Northoff, 2008[22]). In MI, a large number of cardiomyocytes are destroyed and heart function is affected. By replacing these cells, impaired heart function can be compensated and heart output can be improved (Armiñán et al., 2009[2]). There is a strong possibility that if BMSCs are subject to earlier steps of cardiac differentiation *in vitro* prior to transplantation, the engraftment productiveness, as well as the clinical efficiency of treatment, might be enhanced (Antonitsis et al., 2007[1]). Myogenic conversion of BMSCs induced by chemical agents or co-culture with cardiomyocytes has been well explained earlier (Armiñán et al., 2009[2]; Huang et al., 2010[13]). 

Cardiac differentiation is often evaluated by the expression of markers showing a cardiomyocyte phenotype (Armiñán et al., 2009[2]). The previous study showed that the Nkx2.5 gene is expressed as a marker for cardiac mesoderm, cardiomyogenic progenitor cells and continues in cardiomyocytes of embryonic, fetal and adult hearts (Farlie et al., 2017[7]). Hence, the expression of this cardiac transcription factor is an important marker in the analysis of mesoderm development and early cardiomyogenesis (Farlie et al., 2017[7]; Lints et al., 1993[16]). Our results showed that the expression of Nkx2.5 increased on week 2 and then decreased until the endpoint of the experiment in all experimental groups. Given that, this factor plays a fundamental role in the embryological development of the heart and is expressed in the cardiac mesoderm and early cardiomyogenesis stages, such expression pattern would be expected. Our outcome is consistent with other prior studies (Armiñán et al., 2009[2]; Rose et al., 2008[21]).

The previous study showed that GATA4 is normally expressed in early cardiac development specifically in progenitor cells and postnatal growth (Yang et al., 2009[32]). This transcription factor, as well as Nkx2.5, are key players in early heart development by affecting the expression of several encoding cardiac-specific protein genes (Farlie et al., 2017[7]; Yang et al., 2009[32]). Moreover, the cTnT and cTnI are expressed in adult murine hearts, and they regulate muscle contraction in response to intracellular calcium fluctuations (Petropoulou et al., 2017[20]). The CX43 is a type of gap junction proteins that connect neighboring cardiomyocytes; hence, this protein is vital for efficient electrical signals transduction to regulate coordinated contraction of the cardiomyocytes for blood pumping (Yang et al., 2009[32]). β-MHC is the main cardiac thick filament protein that partakes to cardiac muscle contraction (Petropoulou et al., 2017[20]). Although the increased expression of Nkx2.5, GATA4, and CX43 early in week 2 can be a sign of differentiation into cardiac progenitor cells, however, the expression of cTnT, cTnI, CX43, and β-MHC on week 4 can lead us to conclude that BMSCs have differentiated into more mature cardiomyocytes-like with the passage of time. Armiñán et al. (2009[2]) showed that long-term co-culture of BMSCs with neonatal rat cardiomyocytes cause the expression of Nkx2.5, GATA4, and CX43 together with the cardiac-specific markers cTnI, β-MHC, and α-sarcomeric actinin. It has been shown that BMSCs can express cardiomyogenic proteins in co-culture with cardiomyocyte but do not generate functional cardiomyocytes (Siegel et al., 2012[24]). However, another study claimed that co-culture with cardiomyocytes can induce functional myogenic phenotype in BMSCs (He et al., 2010[11]). In contrast, in our study, no beating cells were seen in BMSCs co-cultured with APCs and CNCs in both 2D and microfluidic system for 4 weeks. Considering that BMSCs expressed cardiac-specific factors at week 4, maybe longer co-culture time can lead to more maturation phenotype in these cells. On the other hand, Rose et al. (2008[21]) showed that BMSCs co-cultured with cardiomyocyte can express cardiac proteins like ANF and Nkx2.5 several folds to cardiomyocytes, but they cannot express significantly specific mature cardiomyocyte proteins like α-cardiac actin. These researchers also demonstrated that BMSCs, however, can differentiate into cardiac progenitor cells, but, they retain their primary properties like mesenchymal stromal cell surface markers expression and unexcitable electrophysiological nature (Rose et al., 2008[21]). 

In addition to soluble signaling molecules, direct cell-to-cell interaction is necessary for the differentiation of adult stem cells to cardiomyocytes (Xu et al., 2004[30]). Accordingly, direct co-culture of BMSCs was done with APCs and CNCs in both 2D and microfluidic cell culture systems in this study. We observed a similarity in the differentiation of BMSCs co-cultured with APCs and CNCs into the myogenic lineage. Although in the APCs group, major cells were cardiomyocytes and many fibroblasts were removed, nevertheless the cardiac gene expression of BMSCs co-cultured with both APCs and CNCs groups were similar. It could be due to the secretion of soluble signaling factors from fibroblasts which can compensate for the lower number of cardiomyocytes in CNCs group. Another possible reason may be the communication of fibroblasts with both cardiomyocytes and BMSCs via gap junction proteins like CX43 (Bogdanowicz and Lu, 2013[4]; Kakkar and Lee, 2010[15]; Murthy et al., 2006[19]). 

Another important factor affecting the differentiation of stem cells is shear stress. With produced 10 dyn/cm^2^, the groups in microfluidic chip had higher expression of cardiac genes in comparison with static 2D groups. It can be because of the activation of transcription factors like MEF2C and SMADs that may promote cardiac-specific genes expression (Illi et al., 2005[14]). In this regard, our result revealed that co-culture with APCs and CNCs in both static 2D and microfluidic cell culture systems effectively induced expression of the cardiac progenitor genes Nkx2.5 and GATA4, cardiac-specific genes of β-MHC, cTnI, cTnT as well as gap junction protein CX43 in BMSCs compared with the negative control, as evidenced by increased mRNA levels, however, these results were significantly higher in those in the microfluidic system compared to similar static 2D co-culture groups.

## Conclusion

It was found that, due to produced shear stress, almost all cardiac genes expression were statistically higher in microfluidic condition than static 2D cell culture system. In overall, it can be concluded that fibroblast can compensate the shortage of cardiomyocyte in CNCs group through the secretion of soluble factors. They can also play an interface role between co-cultured cells by transmitting the mechanical forces created by cardiomyocytes to BMSCs. All in all, the differentiation process is a complex process of interrelated phases which can be affected by several environmental cues. Simulation of the cardiac niche with other cues such as mechanical and electrical fields can enhance the final yield of BMSCs maturation into more mature cardiomyocytes with more clinical applicability.

## Funding

This work was supported by grants from the Tehran University of Medical Sciences (grant number 94-04-87-30448).

## Conflict of interest

The authors declare that there is no conflict of interests.

## Figures and Tables

**Table 1:**
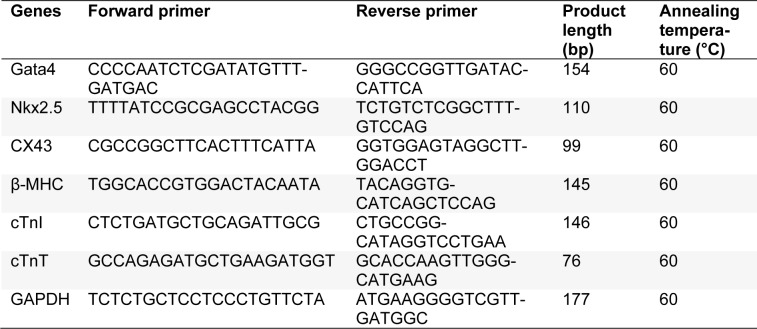
Rat specific real-time PCR primer sequences used in this study
